# Convergence across Tactile Afferent Types in Primary and Secondary Somatosensory Cortices

**DOI:** 10.1371/journal.pone.0107617

**Published:** 2014-09-12

**Authors:** Andrew W. Carter, Spencer C. Chen, Nigel H. Lovell, Richard M. Vickery, John W. Morley

**Affiliations:** 1 School of Medical Sciences, UNSW Australia, Sydney, Australia; 2 Graduate School of Biomedical Engineering, UNSW Australia, Sydney, Australia; 3 Sydney Medical School, University of Sydney, Sydney, Australia; 4 School of Medicine, University of Western Sydney, Penrith, Australia; Emory University, United States of America

## Abstract

Integration of information by convergence of inputs onto sensory cortical neurons is a requisite for processing higher-order stimulus features. Convergence across defined peripheral input classes has generally been thought to occur at levels beyond the primary sensory cortex, however recent work has shown that this does not hold for the convergence of slowly-adapting and rapidly-adapting inputs in primary somatosensory cortex. We have used a new analysis method for multi-unit recordings, to show convergence of inputs deriving from the rapidly-adapting and Pacinian channels in a proportion of neurons in both primary and secondary somatosensory cortex in the anaesthetised cat. We have validated this method using single-unit recordings. The secondary somatosensory cortex has a greater proportion of sites that show convergence of this type than primary somatosensory cortex. These findings support the hypothesis that the more complex features processed in higher cortical areas require a greater degree of convergence across input classes, but also shows that this convergence is apparent in the primary somatosensory cortex.

## Introduction

From the earliest study of the function of somatosensory cortical neurons [1], the preservation of the modality specificity of input classes has become the accepted doctrine. It was subsequently shown that these modalities relate to the different classes of mechanoreceptive afferents. In the glabrous skin of primates and cats, four classes of myelinated mechanoreceptive afferents have been identified [2]: Slowly-adapting type 1 (SA1) afferents, associated with Merkel disk endings; Slowly-adapting type 2 (SA2) afferents associated with Ruffini endings; Pacinian corpuscle afferents (PC), and Rapidly adapting (RA) afferents associated with Meissner corpuscles (or Krause corpuscles in cat). Both SA classes respond to maintained pressure, while RA and PC afferents respond to dynamic stimuli such as a sinusoidal vibration. RA afferents are most sensitive to sinusoidal vibration between 20 and 40 Hz and PC afferents between 100 and 300 Hz [3]. Touch information ascending to cortex remains segregated into these four separate modalities in the dorsal column nuclei [4], [5] and the somatosensory thalamus [6], [7]. Recordings from neurons in primary (S1) and secondary (S2) somatosensory cortex show this same segregation at the level of single neurons [1], [8] and for functional domains in S1 [9], [10].

However, recent evidence suggests that convergence of tactile sensory modalities occurs earlier in the somatosensory pathway. Sakurai et al. [11], using tracing techniques, marked both RA and SA neurons of the mouse vibrissae follicle at the level of brainstem, thalamus, and cortex and found anatomical convergence of RA and SA at all these levels. Pei et al. [12] recorded from peripheral afferents classified as RA or SA due to their response to step indentations. The SA afferents showed a sustained response to the static indentation and no transient response to the removal of the stimulus, whereas RA afferents showed a transient response to the onset and also the offset of stimulation with no static response. Recording from single neurons in S1, Pei et al. found neurons whose response to a step indentation was similar to either an SA or an RA afferent. However, approximately 50% of the S1 neurons they recorded from responded to a step indentation with both a sustained response and a transient off response, suggesting that these neurons received convergent input originating from both SA and RA afferents.

The convergence of RA and SA inputs onto S1 neurons raises the question of whether there is also convergence between the rapidly adapting modalities related to PC and RA afferents. Although this question has not been explicitly addressed, there are reports of RA neurons that show a very broad range of frequency responses, consistent with convergence of RA and PC afferent information in dorsal column nuclei [4], and in S1 [13], [14].

The availability of multi-electrode arrays now allows sampling of large numbers of neural responses simultaneously. In the present study we used a multi-electrode array in S1 and a second array in S2, to simultaneously record multi-unit and single-unit activity in cat cortex. Glabrous skin forelimb pads were stimulated using combinations of high and low frequency vibrations, so as to preferentially activate the separate RA and PC classes of cutaneous afferents. Using a novel analysis technique, we demonstrate that it is possible to show convergence in multi-unit recordings, a method which was validated using single-unit recordings as there was strong agreement between the classifications made using multi-unit and single-unit recordings. The results indicate that although there are many neurons that preserve modality specificity at the level of primary and secondary somatosensory cortex, there is also clear evidence for convergence in both S1 and S2 from RA and PC inputs.

## Materials and Methods

### Ethics Statement

This study was carried out in strict accordance with the recommendations in the Guide for the Care and Use of Laboratory Animals of the National Health and Medical Research Council, Australia. All procedures involving animals were approved and monitored by the University of New South Wales Animal Care and Ethics Committee, project number: ACEC 09/7B. All surgery was performed under anesthesia, and all efforts were made to minimize suffering.

### Animal Preparation

Outbred domestic cats had anaesthesia induced with an intra-muscular dose of ketamine (20 mg/kg) and xylazine (2.0 mg/kg). Anaesthesia was maintained over the three days of an experiment by intravenous infusion of alfaxalone (1.2 mg/kg) delivered in an equal mixture of Hartmann's solution and 5% glucose solution, at approximately 2 ml/kg/hr. The animal received daily doses of dexamethasone (1.5 mg/kg) and a broad spectrum antibiotic (Baytril, 0.1 mL/kg) intra-muscularly, and atropine (0.2 mg/kg) subcutaneously.

A femoral intravenous catheter was inserted for the infusion of anaesthetic, and an intra-arterial catheter for direct monitoring of blood pressure. Tracheostomy was performed, and respiration rate and expired CO_2_ levels were monitored with a Normocap 200 gas analyzer (Datex, Wisconsin, U.S.A.). The animal's core temperature was monitored by means of a rectal thermal probe and maintained with a Physitemp TCAT-2LVB heating pad (Physitemp Instruments Inc., New Jersey, U.S.A.).

The animal was secured in a stereotaxic frame and a craniotomy and durotomy were performed to expose the primary and secondary somatosensory areas. The exposed cortex was mapped by recording evoked potentials using a multichannel recording system (RZ2 TDT, Tucker Davis Technologies Inc., Florida, U.S.A) and an amplifier and headstage (model 1800, AM-Systems, Washington, U.S.A.). Evoked potentials were driven by a vibrotactile stimulus of 2 cycles of 20 Hz sinusoidal indentation with peak-to-peak amplitude of 100 µm. The cortical position of the largest evoked potential for each paw pad was marked on a photograph of the exposed cortex for both S1 and S2.

### Recording and Stimulation

Multi-electrode arrays were inserted into the paw representation regions in S1 and S2 determined from the mapping procedure. In S1, either a 10×10 “planar” array (Blackrock Microsystems, Utah, U.S.A) or 8×8 “linear” array (NeuroNexus, Michigan, U.S.A.) was used, while in S2 only the linear array was used due to the difficulty in accessing the cortical location of S2 with the planar array. Data from these arrays were collected using the RZ2 TDT multichannel recording system through a PZ2 TDT pre-amplifier. Streaming data from up to 96 channels from S1, and 64 channels from S2, were recorded simultaneously without filtering at 12 kHz.

**Figure 1:**
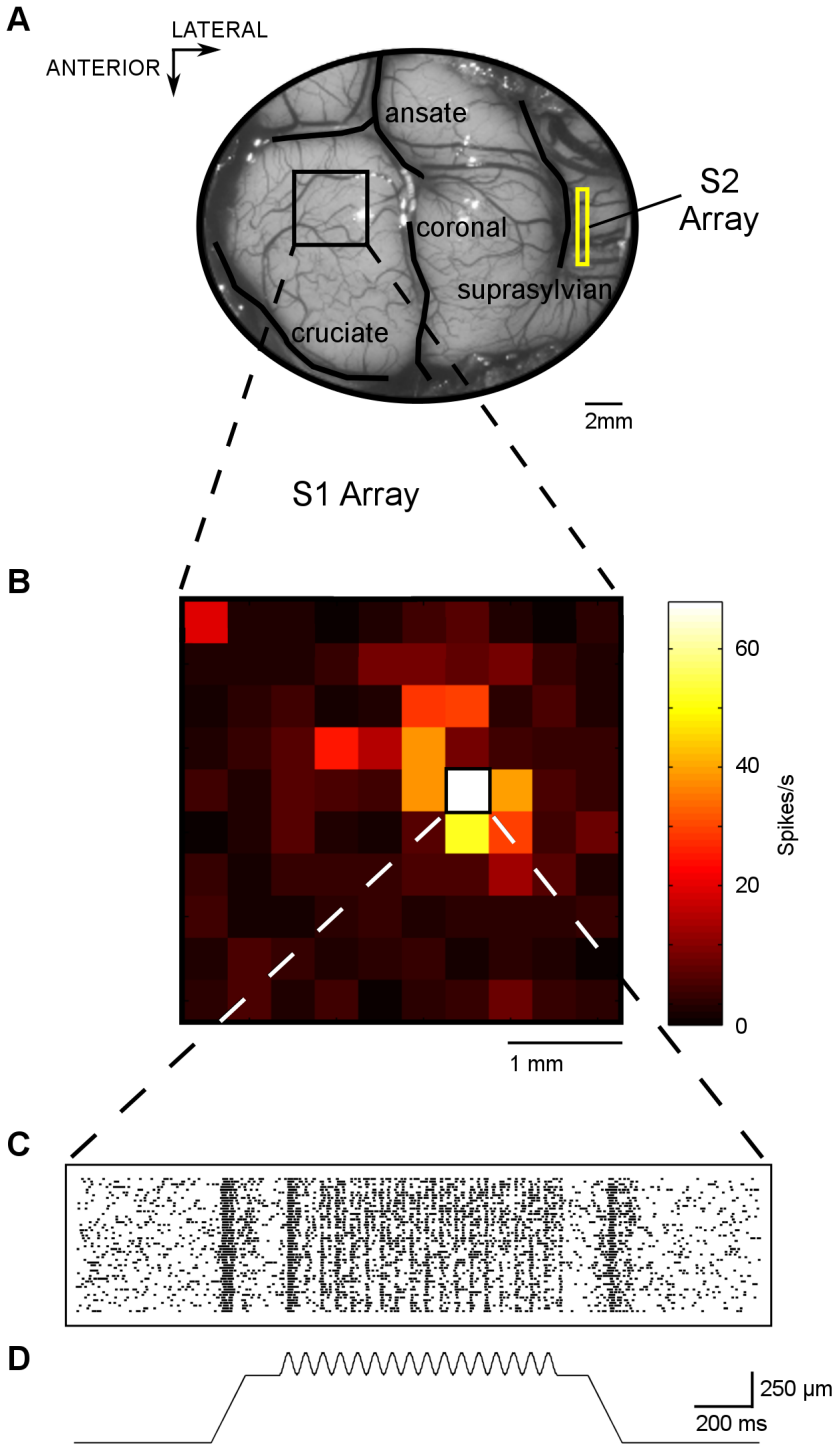
Example stimulus and recording. (A) Photo of anterior parietal cortex with outlines of sulci superimposed. The planar array was inserted into the paw representation region of S1 (black square). A linear array was inserted into S2 region located in the suprasylvian sulci (yellow rectangle). (B) Average baseline-subtracted spike rate for multi-unit activity (MUA) recorded from planar array to the stimulus condition 160 µm at 20 Hz and 16 µm at 200 Hz. Stimulus site is digit 4 of contralateral fore paw. Each of the 100 squares represents the activity on an electrode of the 10×10 planar array. (C) Raster plot of MUA from a single electrode from the planar array for 50 repetitions of stimulus conditions shown in B. (D) Profile of the complex stimulus: 20 Hz + 200 Hz sinusoid — superimposed on a step indentation — aligned with raster plot.

The RZ2 TDT system also drove a Gearing & Watson stimulator and probe with a 5 mm diameter flat perspex tip that was lowered to barely indent the skin of a single paw pad. Hair around the forelimb paw pads was shaved to prevent activation during stimulation. Vibrotactile stimuli were generated as the sum of a low frequency (20 or 23 Hz) and high frequency (200 Hz) sinusoid of variable amplitude, on top of a 500 µm ramp-and-hold indentation. In some animals 23 Hz was used as the low frequency to assess if 200 Hz being a harmonic of 20 Hz had an impact on the neuronal response. Analysis of the data, however, showed no observable difference between the two low frequencies, and so throughout the rest of the paper the low frequency will be referred to as 20 Hz. The ramp onset and offset duration was 100 ms, and there was a 100 ms delay between the ramps and the period of sinusoidal vibration (stimulus shown in Fig. 1D). Stimuli were repeated at 4s intervals. The peak-to-peak amplitudes of the low frequency sinusoid varied from 0 and 160 µm, and the high frequency sinusoid from 0 to 16 µm; these parameters were chosen to activate RA or PC receptors respectively [3]. The amplitudes for the two sinusoids were selected pseudo-randomly for each presentation, and the number of repetitions ranged from 20–60 of each amplitude combination depending on recording session.

### Multi-unit analysis

Data were filtered between 300 and 3000 Hz during post-processing. Common-mode noise across channels of the array was removed through principle component analysis, by removing components identified as common signals across all channels (p<0.05, Student's t-test). Multi-unit spike detection was based on a threshold for each channel set to produce an average pre-stimulus baseline activity of 25 spikes/s for each channel over the 400 ms segments before each stimulus presentation over a recording session. A minimum inter-spike interval of 1 ms was enforced; where multiple spikes within 1 ms were detected, only the spike with the largest peak was retained.

### Response classification

The multi-unit activity (MUA) on each single channel was determined based on the number of detected spikes during the period beginning with the second cycle of the low frequency sinusoid (to discount the onset transient response), and continuing until the last complete period of the low frequency vibration, with a 13 ms allowance for the conduction latency from periphery to cortex.

Analysis of covariance (ANCOVA) was used to identify the rapidly adapting sensory modality subserving each electrode channel by testing for significant covariance of the MUA against: 1. the amplitude of the low frequency vibration; 2. the amplitude of the high frequency vibration; and 3. their interactive combination (facilitative effect).

For tests 1 and 2, ANCOVA was set up with the frequency of interest as a continuous covariate while accounting for the contribution of the other frequency as categorical groups. For test 3, ANCOVA was set up with the interaction term as the covariate while accounting for the marginal contributions by the individual frequencies as categorical groups. Significance (p<0.01) and the sign of the covariance (positive for excitation, negative for inhibition), determined the response classification described in the results.

### Single-unit analysis

Subsequent to the data filtering above, single-unit responses were extracted from the MUA on the basis of a well isolated spike shape in comparison to the background neuronal activity. The single-unit isolation involved three steps. First, large amplitude spikes were isolated from the smaller amplitude spikes for further analysis. Second, a combination of time-voltage window and PCA clustering was used to isolate single-units from the large amplitude spikes. These traditional single-unit discrimination procedures worked best when targeted on the large amplitude spikes, rather than on the entire MUA. Lastly, we obtained a signal-to-noise ratio (SNR) for each of these single-units, and only units with a SNR above 2.75 were used in further analysis [15], [16]. Once these single-units had been isolated, their response was classified according to the same criteria as the MUA outlined above.

The number of neurons contributing to the MUA was estimated by comparing the single-unit activity (SUA) against the surrounding MUA during the vibratory period for each of the stimulus conditions. The MUA was modelled as a multiple of the SUA above a constant baseline. Linear regression was used to estimate the slope between the SUA and the MUA, which we use as the estimate for the number of single-units contributing to the MUA at each corresponding site.

## Results

### Predominance of single-modality response in S1 and S2

The sensory modality of neurons in S1 and S2 was studied in 12 hemispheres from 9 cats by recording MUA from arrays with a linear configuration (Neuronexus array) and planar configuration (Utah array). The linear arrays were an 8×8 penetrating array that recorded data from a vertical cross-section of multiple cortical layers along 1.4 mm of cortex. The planar Utah 10×10 arrays had one hundred 1.5 mm long electrodes, and recorded data from 96 of those electrodes across a 13 mm^2^ horizontal plane of cortex. A total of 2121 classified MUA responses across all electrodes and stimulation sites was obtained (648 from linear array insertions in S1, 491 from planar array insertions in S1, 982 from linear array insertions in S2). Figure 1 shows the cortical insertion sites of the planar and linear array during one recording session (Fig. 1A), the MUA on each of the 96 channels of the planar array in response to a vibratory stimulus presented to digit 4 of the contralateral fore paw (Fig. 1B), and shows rasters from an active channel (Fig. 1C).

**Figure 2:**
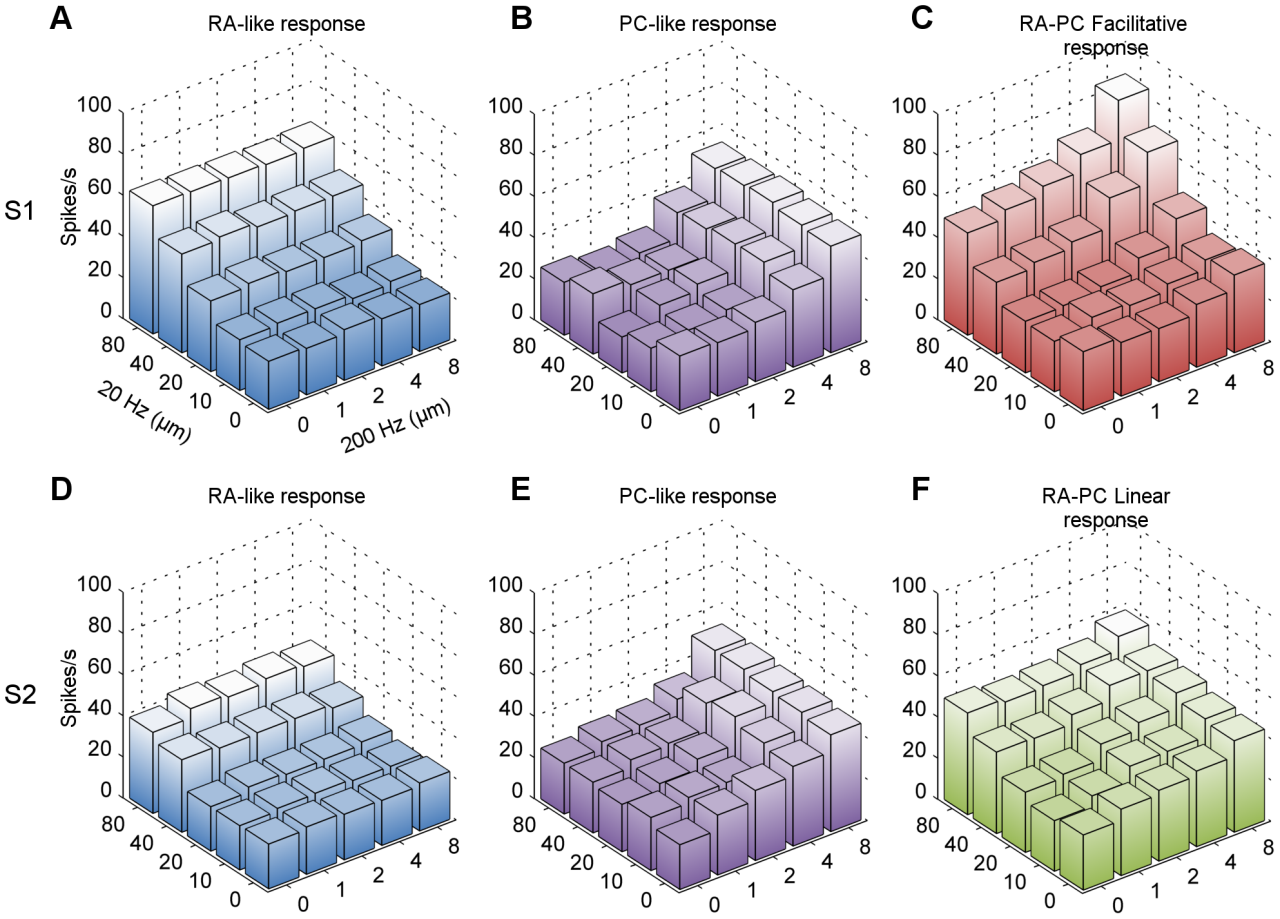
Multi-unit responses and classifications. Each 3D bar graph represents the MUA at one electrode when stimulated with the combinatory 20 Hz + 200 Hz sinusoids. The x-y axes represent the amplitude of the component sinusoids, and the z-axis is the spike rate averaged over the repetitions of the given stimulus condition. The graphs are colour-coded according to their classification: RA (A and D), PC (B and E), RA-PC linear interaction (F), RA-PC facilitative interaction (C). The top row (A, B, and C) are recordings from S1 while the bottom row (D, E and F) are recordings from S2.

The MUA response rate at each channel typically showed strong covariance with the amplitude of the vibratory stimulus. We used this property to classify channels as a *RA-like response* if they showed significant positive covariance of the MUA with the amplitude of the low frequency (20 Hz) sinusoid, but did not show significant covariance for the high frequency (200 Hz) or for the interaction of the frequencies. An example of this class of response from S1 is illustrated in Figure 2A, which plots the MUA for each combination of high and low frequency stimulus amplitudes; comparable data for S2 are shown immediately underneath in Figure 2D. Channels classified as a *PC-like response* showed significant positive covariance of the MUA with the amplitude of the high frequency (200 Hz) sinusoid, but did not show significant covariance for the low frequency or interaction of the frequencies (Fig. 2B and 2E, S1 and S2 respectively).

### Cross-frequency interactions in multi-unit data indicating modality convergence

Both S1 and S2 had MUA driven strongly by both low and high frequency vibration. These channels showed significant positive covariance to both the low and high vibration frequencies, and if this occurred without significant covariance in the interaction of the frequencies, we classified these channels as *RA-PC linear interaction* (Fig. 2F, data from S2). Channels that showed significant positive covariance in the high frequency, low frequency and interaction tests were classified as *RA-PC facilitative interaction* (Fig.2C, data from S1). Occasionally, channel recordings showed negative covariance with vibration amplitude, indicative of inhibition rather than excitation; these represented less than 10% of all recordings, and are not reported on further in this paper.

**Figure 3:**
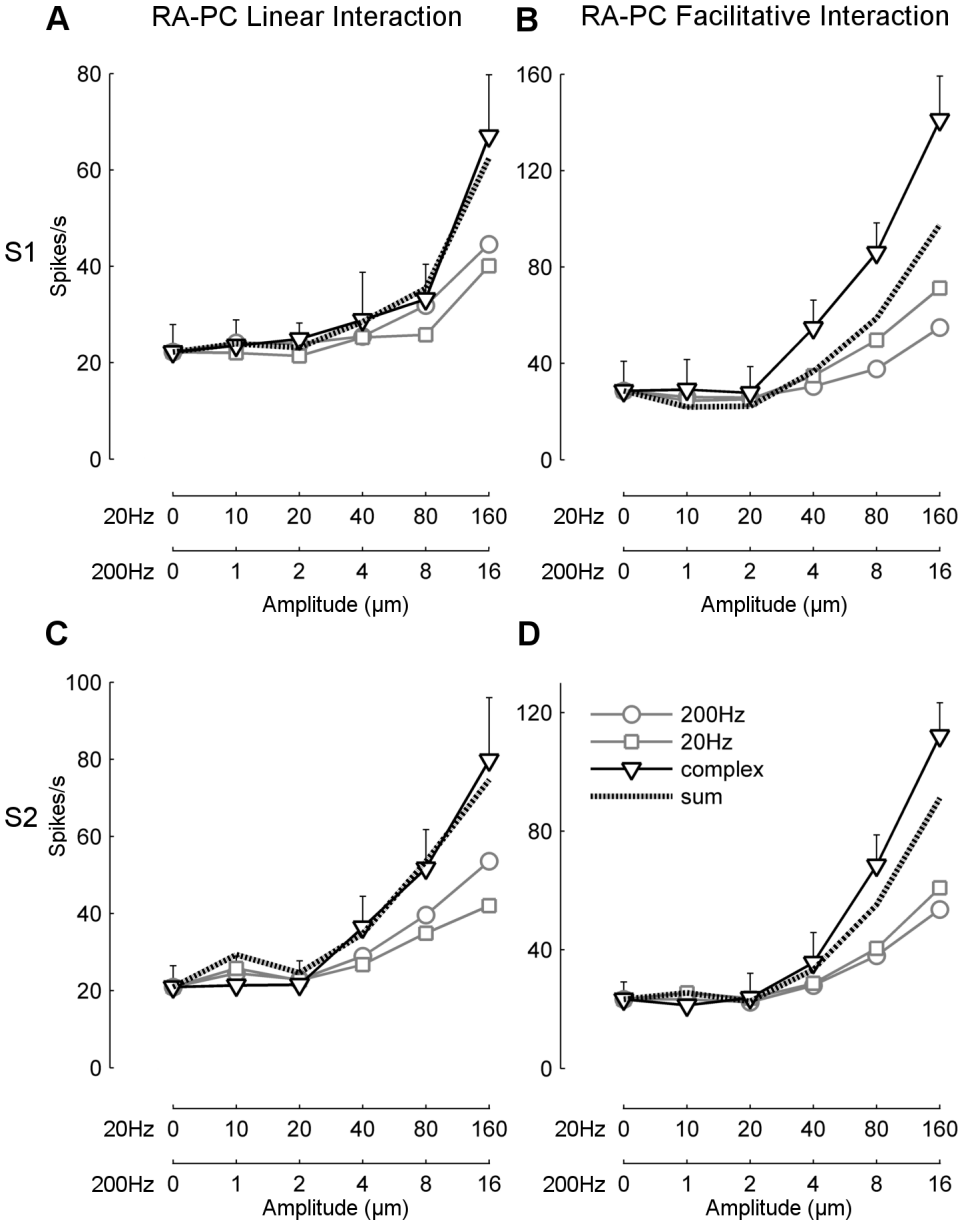
RA-PC linear and facilitative interactions. Average spike rate of multi-unit activity from individual channels exhibiting RA-PC linear interaction or RA-PC facilitative interaction. The stimulus conditions plotted are pure 20 Hz sinusoids (grey square), pure 200 Hz sinusoids (grey circle), and the simultaneous combination of 20 Hz and 200 Hz (black triangle). The response to the combined stimulus is compared to the baseline-subtracted summed response from the pure 20 Hz and pure 200 Hz stimulus (black dotted line). Error bars denote standard deviation.

The MUA is the combined response of multiple cortical neurons, and so the channels classified as RA-PC linear interaction may represent summed activity from RA-like and PC-like neurons. The response to the dual frequency stimulus was modelled as the arithmetic sum of the responses to the pure 200 Hz sinusoid (Fig.3A, circles) and the pure 20 Hz sinusoid (Fig. 3A, squares) and is shown by the dashed line in Fig. 3A. This model is a good fit to an actual response classified as RA-PC linear interaction when the two sinusoids were presented simultaneously across a range of amplitude combinations (Fig. 3A, triangles). Examples of this form of response were found in both S1 (Fig. 3A) and in S2 (Fig. 3C). For the RA-PC facilitative interaction class (Fig. 3B & D, for S1 and S2 respectively) it is clear that the arithmetic sum (dashed line) is substantially less than the response to combined stimulation with the two sinusoids (triangles). This demonstrates that the responses of this class cannot simply be due to recording mixed activity from pure RA-like and PC-like individual neurons.

**Figure 4:**
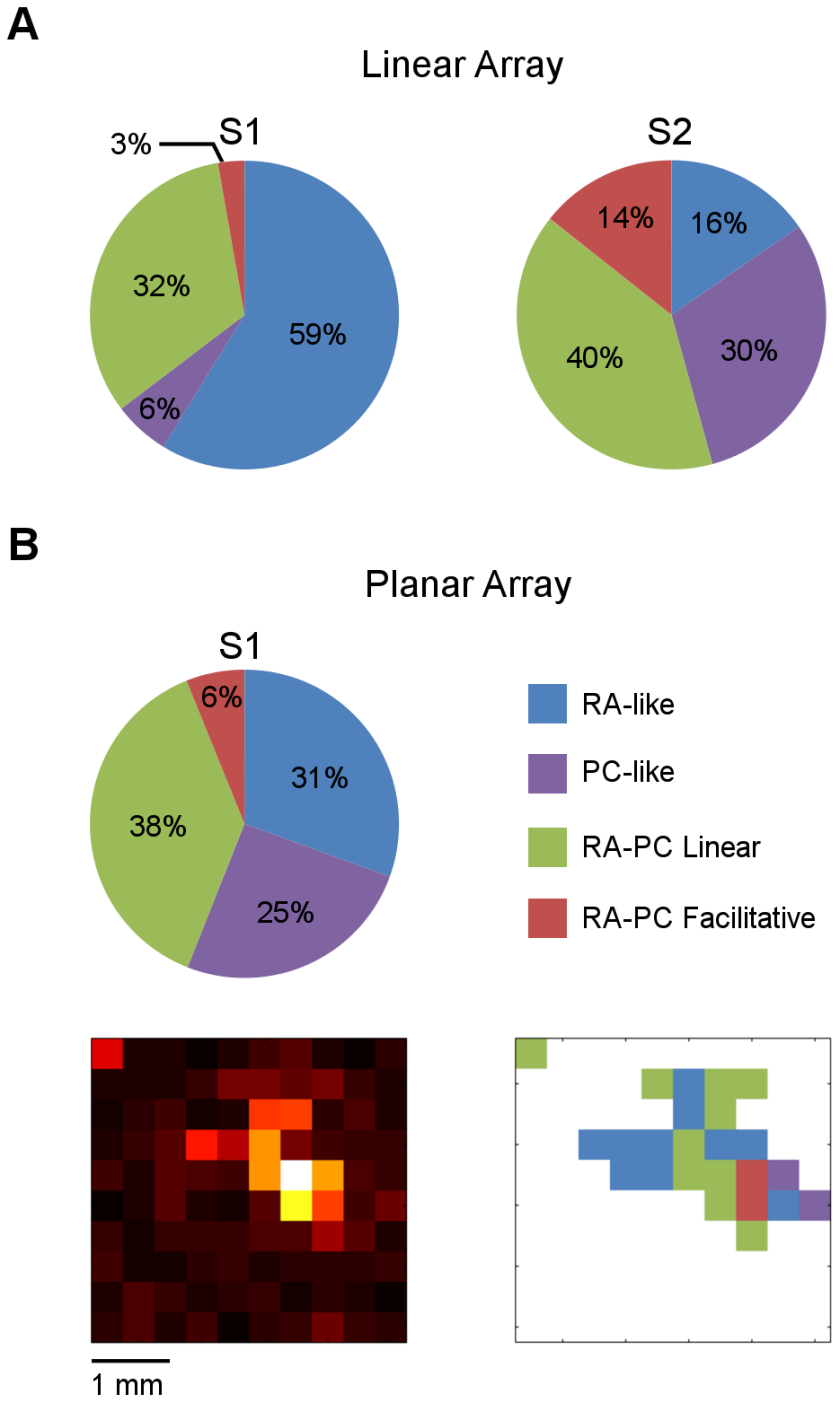
Proportions of RA-PC response classes. (A) The distribution of channels in RA-like, PC-like, RA-PC Linear Interaction and RA-PC Facilitative Interaction classes found using the linear arrays in S1 (left) and S2 (right) across all responsive channels and stimulus sites. (B) The distribution of classes found using the planar array in S1 (top left), the averaged baseline-subtracted activity recorded by the planar array from one animal (bottom left), and the spatial organization of these classes (bottom right) for this given recording. White represents unresponsive channels.

The proportions of channels categorized into these four response classes are illustrated in Figure 4. The top graphs (Fig. 4A) are based on data obtained with the linear arrays, and show that S2 had significantly greater response to high frequency vibration than S1, shown in the proportion of all three response categories containing a PC-like contribution (84% in S2 compared with 41% in S1, p<0.01, Chi Square). The S1 recordings with the linear array may be biased in favour of RA-like responses as the insertion site was determined using a low frequency search stimulus. The planar array data from S1 (Fig. 4B) is shown for comparison as it samples a much larger cortical area. The planar array data shows larger proportions of all the classes with a PC-like contribution when compared to the linear array data from S1 (25% : 6% PC-like, 38% : 32% RA-PC linear, 6% : 3% RA-PC facilitative). The spatial distribution across the activated region of S1 of these four response classes is shown at the bottom of Figure 4B for the data recorded with a planar array in one hemisphere. The white background indicates channels that were not significantly activated at this stimulus site.

### Cross-frequency interactions in single-unit data indicating modality convergence

**Figure 5:**
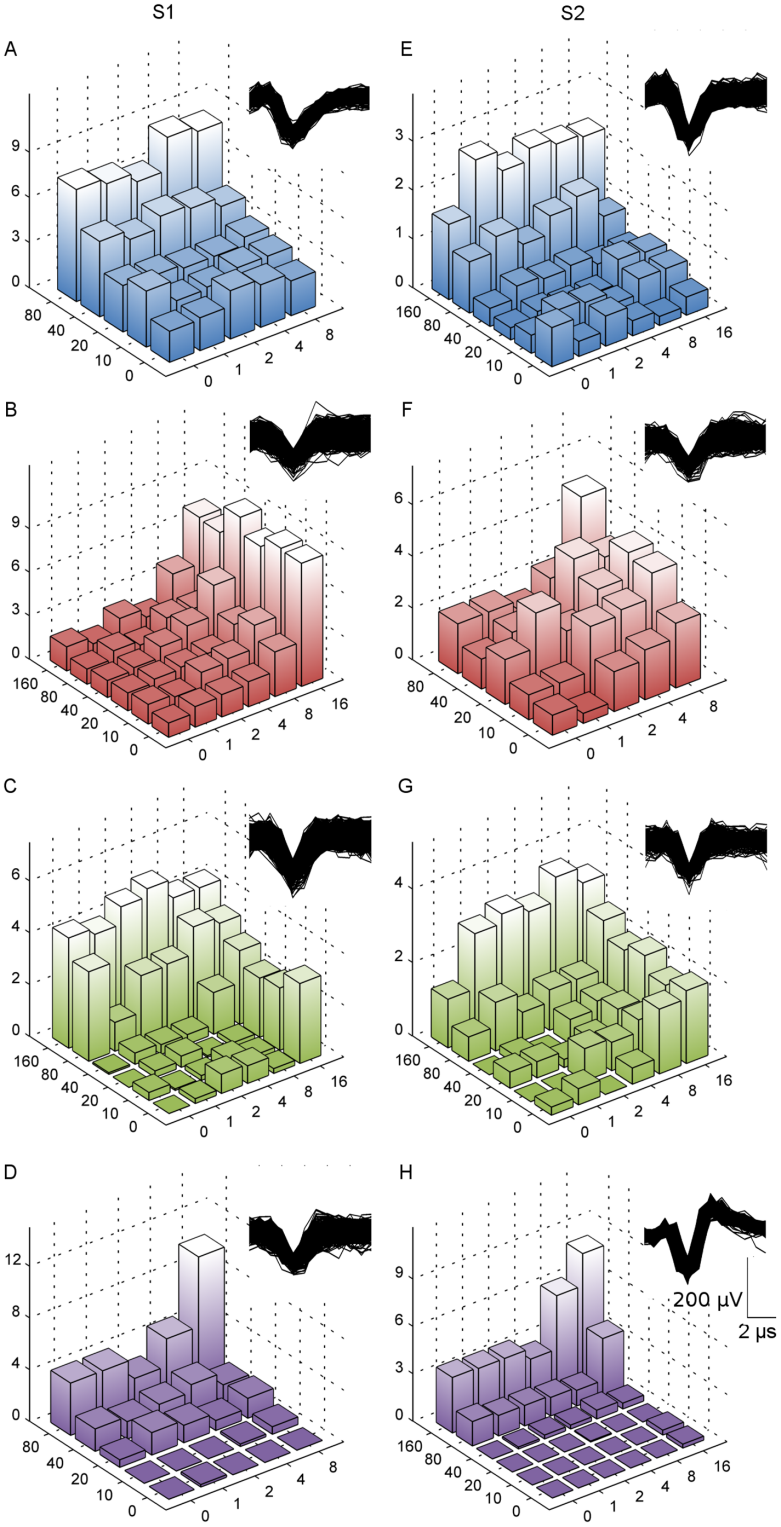
Single-unit responses and classifications. Each 3D bar graph represents the SUA for the spike shown in each corresponding inset when stimulated with the combinatory 20 Hz + 200 Hz sinusoids. The x-y axes represent the amplitude of the component sinusoids, and the z-axis is the spike rate averaged over the repetitions of the given stimulus condition. The graphs are colour-coded according to their classification: RA (A and E), PC (B and F), RA-PC linear interaction (C and G), RA-PC facilitative interaction (D and H). The left examples (A, B, C and D) are recordings from S1 while the examples on the right (E, F and G) are recordings from S2.

**Figure 6:**
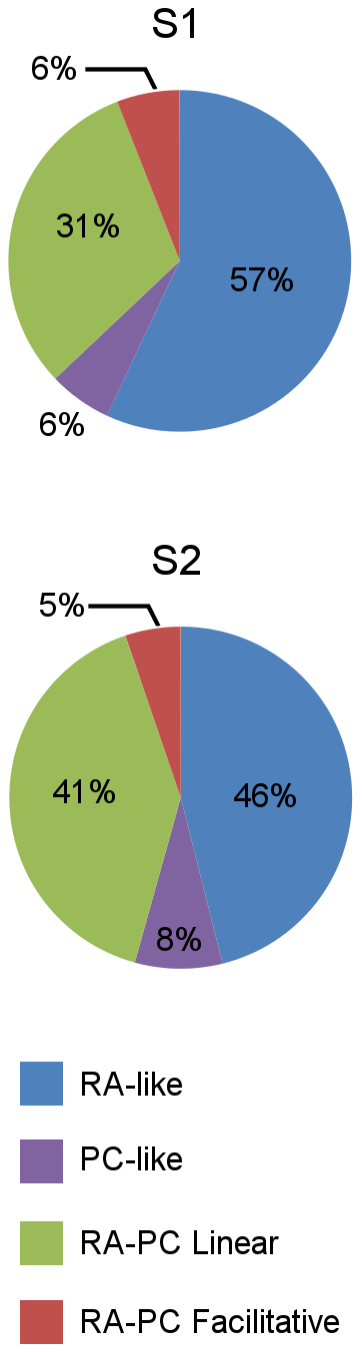
Proportions of RA-PC response classes in single-unit data. The distribution of isolated single-units in RA-like, PC-like, RA-PC Linear Interaction and RA-PC Facilitative Interaction classes found in S1 (top) and S2 (bottom) across stimulus sites.

We confirmed the convergence of these response classes by isolating single-units from the recorded MUA. A total of 516 stimulus-driven single-units from both S1 (267 units) and S2 (249 units) were isolated. All classes identified in the MUA were also found to be present using single-unit data. Figure 5 shows single-unit examples of the 4 classes identified in the MUA from both S1 and S2: RA-like (A & E), PC-like (B & F), RA-PC Linear (C & G) and RA-PC Facilitative (D & H). The proportions of these classes found using single-units is shown in Figure 6, and are generally a broad match to those for MUA classification, although S1 shows a closer match than S2. The single-unit response was classified as the exact same type as the surrounding MUA in 59% of comparisons in S1 and 62% in S2 e.g. RA-like single-unit within RA-like MUA. In the remaining comparisons the SUA was of a different classification to the surrounding MUA. The MUA that showed a response to both frequencies may be due to a mixture of pure frequency responsive cells and also convergent cells, while the MUA that was responsive to only the low or the high frequency may be predominantly composed of cells only responsive to that single frequency range. When we restricted comparisons of SUA and MUA to those that responded to only low or high frequency, there was very close agreement between the SUA and MUA classification with S1 showing 98% match between single-unit response type and surrounding MUA response type and the match in S2 was 97%.

### Single-unit contribution to MUA

We estimated the number of driven neurons around an electrode in our MUA by assuming a linear relationship between the single-unit spike rate and the MUA from which it was extracted (see Materials and Methods). The median slope from this linear fitting was 7, which we took as the average number of neurons contributing to any given multi-unit response, with a lower and upper quartile of 3 and 19 respectively.

## Discussion

### Novel method of assessing convergence of sensory modalities in multi-unit recordings

The data presented in this paper were all obtained with large multi-electrode arrays, and are primarily based on multi-unit recordings that originate from a number of single-unit responses recorded at each electrode. Our isolated single-units typically accounted for between 5–33% of the activity in a multi-unit recording, suggesting that most of our multi-unit activity is derived from 3–19 active neurons. In general the properties of multi-unit recordings made in somatosensory cortex are similar to those of single-units in terms of receptive field location and mean spike rate [14], [17]. The same assumption can not be made with regard to convergence, as a multi-unit recording may be driven by both low frequency and high frequency vibration, but this may simply reflect the activity of two or more single-units contributing to that multi-unit recording, each of which is purely responsive to either the low or high frequency vibration. To demonstrate convergence in multi-unit recordings, we have used a novel stimulus paradigm and analysis technique of summing simple 20 Hz and 200 Hz sinusoids into a complex stimulus and analysing the component responses. We found response properties with the complex stimulus that were not found when the responses to the simple stimuli components were summed, which can only be due to convergence of these simple inputs onto common neurons contributing to our recording, as shown in the RA-PC facilitative interaction in Figure 3B & D. This approach likely underestimates the degree of convergence, as it can not account for neurons that receive convergent input but whose response is little different from the summed response to the two separate components. We isolated several single neurons that showed convergent input from both 20 and 200 Hz, but whose response to simultaneous combined stimulation was not distinguishable from a linear sum of the response to the pure sinusoids. This indicates that some proportion of our MUA classified as RA-PC linear interaction likely represent true convergence onto single neurons, and so the estimate of convergence based on the proportion of RA-PC facilitative interaction represents a lower bound on the convergence of these classes.

### Convergence of PC and RA

Since Mountcastle's 1957 [1] paper, it has been accepted that neurons in S1 preserve modality-specificity based on their peripheral receptor input. This observation was extended to area S2 in the cat [8] and primate [18]. We have now demonstrated that there is convergence of input deriving ultimately from RA and PC afferents onto single neurons in both S1 and S2. The failure of previous studies to report evidence of cross-modal convergence of RAs and PCs may be due to different definitions of what constitutes PC afferent input. The definition of PC input used in this paper relies on the specificity of this afferent class for low amplitude high frequency vibration, determined by MUA that covaried with amplitude changes. In contrast, Burton and Sinclair [18] required neurons in S2 to show 1∶1 entrainment, which for 200 Hz stimulation requires 200 spikes/s. Such a response rate in the somatosensory cortex is rarely observed, for instance Yau et al. [19] using a non-vibrating but highly salient stimuli in area 2 of S1 reported response rates of only 12 to 29 spikes/s. Entrainment aside, a closer examination of the data of Burton and Sinclair [18] shows evidence of cells that appear to display convergent input from RA and PC afferents (e.g. Fig. 1C and 5A both show cells with strong amplitude modulation at both low (10 or 30 Hz) and high (300 Hz) frequency).

The failure of previous studies to report evidence of cross-modal convergence of RAs and PCs may also be due to sampling limitations of traditional electrophysiological studies. Using multi-electrode arrays and MUA analysis, our methods permit us to sample with 64 or 96 electrodes, each electrode recording simultaneously from approximately 3 to 19 neurons. This represents a significant sample of the cortical activity which is essential when dealing with a population that is often non-responsive [18] and where the population being sought represents only a small proportion of the total.

### Comparison of response types between S1 and S2

The proportions of RA-like and PC-like responses recorded in S1 and S2 with the linear array differ between the two regions, with S1 displaying a greater proportion of channels classified as RA-like than S2 (59% compared to 16%), and S2 a greater proportion classified as PC-like than S1 (30% compared to 6%). The planar array recordings show a much less exaggerated difference, but still maintain this S1-S2 difference of more RA-like and less PC-like with S1 having 31% RA-like and 25% PC-like. This difference between the two regions, with S1 being more RA-like dominant and S2 being more PC-like dominant, is consistent with previous reports [8], [18], [20]–[24].

S2 contains a greater proportion of channels showing a response classed as RA-PC facilitative interaction compared with S1 (14% to 3% (linear array) or 6% (planar array)). This could reflect the hierarchical relationship between the two regions [25], [26], with S2 being higher in the processing hierarchy and having a greater proportion of its neurons integrate input from multiple sources. Additionally a hierarchical relationship implies that the proportion of convergence in S2 already includes the convergent inputs observed in S1. An alternative explanation is that S2 simply has more PC inputs, and so we might expect to record a correspondingly higher level of convergence [21]. Comparing the ratio of MUA showing RA-PC facilitative response to PC-like response for both S1 and S2, both ratios are approximately equal to each other in the two regions (0.5 for both regions), suggesting that the higher proportion of RA-PC facilitative responses we find in S2 is likely due to the larger proportion of PC-like responses in S2 compared with S1.

## Conclusion

Tactile exploration of an object will activate all classes of mechanoreceptive afferent, and forming a complete mental image of the object will require integration of information across these various afferent types. While each type of afferent maintains segregated channels enroute to the brain, we have shown for the first time that modality specificity of inputs deriving from PC and RA afferents is not fully maintained in either S1 or S2 due to cross-modal convergence onto common neurons. We were able to demonstrate this convergence using a novel analysis of multi-unit activity from large multi-electrode arrays, validated with single-unit data.
